# Cardiovascular extracellular microRNAs: emerging diagnostic markers and mechanisms of cell-to-cell RNA communication

**DOI:** 10.3389/fgene.2013.00214

**Published:** 2013-11-12

**Authors:** Virginie Kinet, Julie Halkein, Ellen Dirkx, Leon J. De Windt

**Affiliations:** Department of Cardiology, Faculty of Health, Medicine and Life Sciences, Cardiovascular Research Institute Maastricht School for Cardiovascular Diseases, Maastricht University Maastricht, Netherlands

**Keywords:** extracellular microRNA, inter-cellular communications, biomarkers, cardiovascular diseases

## Abstract

Cardiovascular diseases are a leading cause of morbidity and mortality in Western societies. It is now well established that microRNAs (miRNAs) are determinant regulators in various medical conditions including cardiovascular diseases. The recent discovery that miRNAs, while associated with different carriers, can be exported out of the cell, has triggered a renewed interest to analyze the potential to use extracellular miRNAs as tools for diagnostic and therapeutic studies. Circulating miRNAs in biological fluids present a technological advantage compared to current diagnostic tools by virtue of their remarkable stability and relative ease of detection rendering them ideal tools for non-invasive and rapid diagnosis. Extracellular miRNAs also represent a novel form of inter-cellular communication by transferring genetic information from a donor cell to a recipient cell. This review briefly summarizes recent insights in the origin, function and diagnostic potential of extracellular miRNAs by focusing on a select number of cardiovascular diseases.

## INTRODUCTION

Cardiovascular disease is a global health problem. Of all 60 million cases of deaths from all causes worldwide in 2005, an estimated 18 million were due to cardiovascular diseases, three times more than caused by infectious diseases including HIV/AIDS, tuberculosis, and malaria combined (World Health Organization, 2013). Current clinical diagnostics fail to identify early changes of adverse cardiac or vascular remodeling, forcing clinicians to wait for these cardiovascular disorders to become clinically evident before initiating intervention. Additionally, treatment efficacy cannot be reliably assessed in individual patients, in part as many interventions are merely treating symptoms (e.g., diuretics). Ideally, one would not assess intervention success based on survival or hospitalization, but build in intermediate end-points that can reliably assess therapeutic benefit. Thus, for the cardiovascular field, there is a need to identify intermediate diagnostic measures that monitor subtle biological changes in the heart or vasculature that directly reflect and predict adverse changes before they become clinically apparent.

To achieve the goal of early diagnosis and treatment, microRNAs (miRNAs) could play an unexpected role. MiRNAs are a group of non-coding regulatory RNAs of about 22 nucleotides that control gene expression at the post-transcriptional level (Bartel, 2004) and act as crucial regulators of most physiological and pathological processes. Indeed, dysregulation of intracellular miRNA expression has been linked to many clinically relevant cardiovascular conditions (Small and Olson, 2011; Da Costa Martins and De Windt, 2012; Gladka et al., 2012; van Empel et al., 2012). Unexpectedly, the recent discovery of circulating miRNAs has opened the possibility to study this class of biologically active agents as modes of inter-cellular information flow as well as biomarkers of disease. Here, we present an overview of the different carriers associated with extracellular miRNAs that render them stable in biological fluids, present the current level of understanding of their role in cell-to-cell communication and give an overview about the clinical utility of extracellular miRNAs as putative biomarkers for cardiovascular disease entities.

## VEHICLES THAT STABILIZE EXTRACELLULAR miRNAs

**FIGURE 1:**
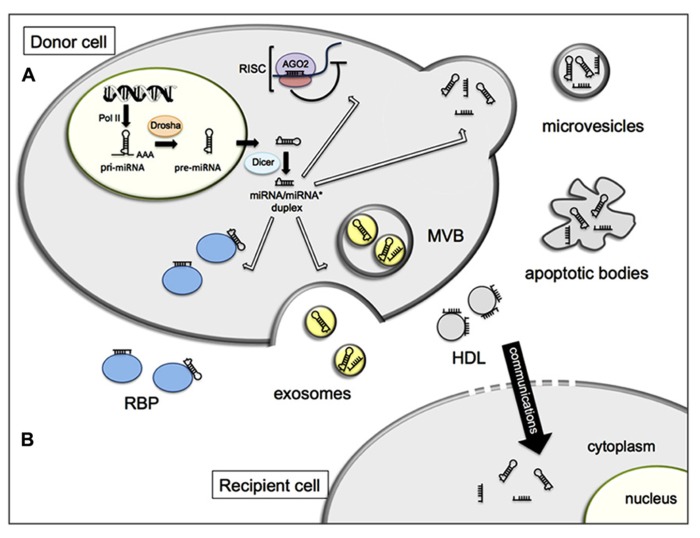
**Schematic representation of cellular release (A) and inter-cellular communication (B) of miRNAs. (A)** In the nucleus, miRNA genes are mainly transcribed by the RNA polymerase II (Pol II) into primary miRNAs (pri-miRNAs) and processed to precursor miRNAs (pre-miRNAs) by the Drosha complex. Pre-miRNAs are exported to the cytoplasm and cleaved by Dicer to produce a double stranded miRNA duplex. The duplex is separated and a mature miRNA is incorporated into the RNA-induced silencing complex (RISC) while the other strand is likely subject to degradation. Within the RISC complex, miRNAs bind to their target messenger RNAs (mRNAs) to repress their translation or induce their degradation. In addition, miRNAs can be exported out of the cells and transported by various carriers, membrane-derived vesicles (exosomes, microvesicles, apoptotic bodies), miRNA-binding protein complexes (RBP), or high density lipoproteins (HDL). **(B)** Extracellular miRNAs can be transferred to recipient cells where they alter gene expression.

The first accounts of extracellular miRNA biomarkers were described in serum of lymphoma patients (Lawrie et al., 2008) and in plasma and serum of prostate cancer patients (Mitchell et al., 2008). Subsequently, it became evident that miRNAs can be exported from cells, and found in most extracellular biological fluids including plasma, serum, saliva, urine, tears, and breast milk (Chim et al., 2008; Weber et al., 2010; Boon and Vickers, 2013). Extracellular miRNAs are unexpectedly stable, and must be shielded from degradation, as naked RNA is readily targeted by exonucleases that are abundantly present in various extracellular fluids (Kamm and Smith, 1972). Indeed, miRNAs are packaged in microparticles (exosomes, microvesicles, and apoptotic bodies; Valadi et al., 2007; Hunter et al., 2008; Zernecke et al., 2009) or by their association with RNA-binding proteins including Argonaute 2 (Ago2; Arroyo et al., 2011) or lipoprotein complexes such as high-density lipoprotein (HDL; Kamm and Smith, 1972; Vickers et al., 2011; **Figure 1**).

The term exosomes was used for the first time in 1981 to describe exfoliated membrane vesicles (Trams et al., 1981). Exosomes are small (40–120 nm) extracellular microvesicles arising from multivesicular bodies (MVBs) and released by exocytosis of these MVBs (Heijnen et al., 1999). They are produced by a variety of cells including epithelial cells (Zhou et al., 2011), hematopoietic cells (Laulagnier et al., 2004), endothelial cells (Halkein et al., 2013), and tumor cells (Mitchell et al., 2008). Exosomes have also been identified in most circulating body fluids such as plasma, urine, milk, saliva, and sperm (Thery et al., 2006). The interest of exosome biology was increased following the demonstration that exosomes can serve as carriers for miRNAs (Valadi et al., 2007; Gallo et al., 2012). Selection processes must take place of miRNA uploading into exosomes, as some miRNAs can be either more or less expressed in donor cells or in the secreted exosomes (Valadi et al., 2007; Pigati et al., 2010), suggesting the existence of cellular mechanisms that actively concentrate specific miRNA species in exosomes (Valadi et al., 2007; Pigati et al., 2010).

Microvesicles or shedding microvesicles (SMVs) are another form of small, defined vesicles (Pant et al., 2012) that are shed from the plasma membrane by a wide variety of cells (Heijnen et al., 1999). They are larger (0.1–1 μm) than exosomes (Heijnen et al., 1999) and their mechanism of production is also different. While exosomes are produced by exocytic fusion of MVBs, microvesicles are produced by budding of vesicles from the plasma membrane (Mathivanan et al., 2010). The presence of miRNAs in microvesicles were described for the first time in 2008 (Hunter et al., 2008).

A final vesicular form where miRNAs reside are apoptotic bodies or apoptotic blebs, byproducts of apoptotic cells. Apoptotic or dying cells release membrane vesicles into the extracellular environment via bleeding of the plasma membrane (Mathivanan et al., 2010). These are larger particles (1–5 μm) with heterogeneous shape (Gyorgy et al., 2011). In atherosclerotic vascular disease, endothelial cells can produce apoptotic bodies enriched with miR-126. These endothelial cell-derived apoptotic bodies trigger, via miR-126, the production of CXC chemokine CXCL12 in the recipient vascular cells which limits atherosclerosis and confers plaque stability (Zernecke et al., 2009).

Apart from packaging miRNAs in cell-derived vesicles, a significant fraction of extracellular miRNAs is associated with RNA binding proteins, including nucleophosmin (NPM1), that provide protection from degradation (Wang et al., 2010b). It was also demonstrated that many extracellular miRNAs are bound to proteins of the Argonaute family, primarily Ago2, although additional members such as Ago1, Ago3, and Ago4 might be also associated with miRNAs (Arroyo et al., 2011; Turchinovich et al., 2011). These latter studies are at odds with the findings by Gallo et al. (2012). This discrepancy may arise from the different protocols used for microvesicle- and RNA-isolation and subsequent data normalization, emphasizing the need for further protocol standardization (Turchinovich et al., 2012). Finally, it was recently shown that extracellular miRNAs can be transported by HDL (Vickers et al., 2011; Norata et al., 2013). Whereas vesicle carriers are composed of a bilayer of phospholipids, lipoproteins have a single layer of lipids (Boon and Vickers, 2013).

## EXTRACELLULAR miRNAs IN CELL-TO-CELL COMMUNICATION

Interestingly, extracellular miRNAs also present a newly discovered potential of intercellular communication. It is now established that transfer of genetic information in the form of RNA exists (Valadi et al., 2007) and that this form of transfer between cells is of functional relevance by exerting gene silencing in the recipient cells (Kosaka et al., 2010; Mittelbrunn et al., 2011; Halkein et al., 2013; **Figure 1**). While the biological mechanisms driving the secretion of miRNAs are still under debate (Kosaka et al., 2010), this newly discovered manner of genetic exchange between cells opens a new aspect of how adjacent cells within an organ may communicate and how a miRNA can affect a cell type or a tissue where it is not produced. Since the first discovery of the extracellular miRNAs as intercellular communicators, this field of research is still growing. Increasing evidence suggests that this form of communication occurs in various physiological processes such as the regulation of the immunity (Mittelbrunn et al., 2011) or cellular migration (Zhang et al., 2010), but also participates in pathological situations including tumor development (Yang et al., 2011).

For cardiovascular diseases, only three examples of intercellular miRNA communication have been demonstrated. The first study presented evidence that endothelial cell-derived apoptotic bodies are generated during atherosclerosis and lead to the induction of the expression of CXCL12 in recipient endothelial cells. These endothelial cell-derived apoptotic bodies also induce the recruitment of progenitor cells in mice with atherosclerosis and reduce the extent of plaque formation. It was finally demonstrated that the atheroprotective effects of endothelial apoptotic bodies are mediated by miR-126 (Zernecke et al., 2009). Additionally, shear stress as well as the shear-responsive transcription factor Kruppel-like factor 2 (KLF2) induces the expression of the cluster miR-143/145 in endothelial cells and also its enrichment in extracellular vesicles produced by the treated-endothelial cells. It was demonstrated that these endothelial-derived miR-143/145-containing vesicles are transferred to smooth muscle cells and induce an atheroprotective phenotype in recipient cells. MiR-143/145 from endothelial cells repress target genes in recipient smooth muscle cells such as ELK1 and KFL4 implicated in smooth muscle cell fate and plasticity (Hergenreider et al., 2012).

More recently, it was demonstrated that the anti-angiogenic fragment 16K prolactin (PRL) positively regulates the expression of miR-146a in endothelial cells where it affects mainly the cell survival and proliferation by down-regulating NRAS gene expression. Even more, the treatment of endothelial cells with 16K PRL also increases miR-146a level in the exosomes secreted by the donor endothelial cells. There is an uptake of the endothelial cell-derived exosomes by cardiomyocytes and transferred miR-146a reduces the metabolism of the recipient cells. This model was proposed to play a central role in the development of peripartum cardiomyopathy since blocking miR-146a activity attenuated the disease in mice (Halkein et al., 2013).

The use of exosomes as therapeutic vehicles should now also be considered. In the field of cardiovascular diseases, a first study has presented the potential of cardiomyocyte progenitor cells-derived exosomes to stimulate endothelial cell migration in the treatment of myocardial infarction (MI) (Vrijsen et al., 2010).****More recently, *in vivo *delivery of cardiac progenitor-derived exosomes has been shown to inhibit cardiomyocyte apoptosis in a mouse acute ischemia/reperfusion model (Chen et al., 2013). In the context of therapeutics, the first report is now also available demonstrating that cells can be engineering to express specific ligands at the surface of the exosomes and load these carriers with therapeutic siRNA species (Alvarez-Erviti et al., 2011). Additional efforts for a better understanding of the mechanisms of extracellular miRNA secretion and the targeting of recipient cells by microvesicles are expected in the future.

### CIRCULATING miRNAs AS BIOMARKERS OF CARDIOVASCULAR DISEASES

Circulating B-type natriuretic peptide (BNP) and its amino-terminal fragment, N-terminal pro-brain natriuretic peptide (NT-proBNP) are clinically established as diagnostic biomarkers for heart failure (Januzzi et al., 2006). For patients with acute myocardial infarction (AMI), circulating levels of cardiac troponins (cTns) are considered a gold standard for the early diagnosis of this disease (Jaffe et al., 2000). Unfortunately, elevated levels of cTn concentrations have also been reported in patients with end-stage renal disease (Collinson et al., 1998), which indicates that this marker lacks specificity for AMI. For atherosclerosis, many biomarkers have been proposed, such as C-reactive protein, interleukins IL-1 and IL-6, apolipoproteins apoA-I and apoB, and fibrinogen (Kampoli et al., 2009). It is not clear whether these new biomarkers are useful predictors of future cardiovascular events. Therefore, it remains essential to continue to explore new biomarkers with even greater discriminatory power for the various subtypes of heart disease. In recent years, several studies have reported on the use of miRNAs as circulating biomarkers for diagnosis or prognosis of various human diseases including cardiovascular diseases (Salic and De Windt, 2012; **Table 1**).

**Table 1:** Extracellular miRNAs as biomarkers in cardiovascular diseases.

miRNA biomarkers	Diseases	Source	Regulation	Correlation	Design of study	Reference
miR-423-5p	HF	Plasma	Up	proBNP	30 HF; 20 non-HF with dyspnea; 39 healthy subjects	Tijsen etal. (2010)
miR-18b-3p						
miR-129-5p						
miR-1254						
miR-675						
miR-622						
miR-126	HF	Plasma	Down	BNP	10 HF; 17 asymptomatic control	Fukushima etal. (2011)
miR-208b	AMI	Plasma	Up		32 AMI; 36 non-AMI with AP	Corsten etal. (2010)
miR-499						
miR-1	AMI	Plasma	Up	QRS widening	93 AMI; 66 healthy subjects	Ai etal. (2010)
miR-1	AMI	Serum	Up	CK-MB	31 AMI; 20 healthy subjects	Chen etal. (2013)
miR-133a	STEMI	Serum	Up		216 STEMI	Eitel etal. (2012)
miR-499	AMI	Plasma	Up		14 AMI; 15 congestive HF; 10 healthy subjects	Adachi etal. (2010)
miR-1	AMI	Plasma	Up		33 AMI; 33 non-AMI with other cardiac diseases; 10 healthy subjects	Wang etal. (2010a)
miR-133a						
miR-499						
miR-208a						
miR-30a	AMI	Plasma	Up		18 AMI; 30 healthy subjects	Long etal. (2012)
miR-195						
let-7b						
miR-126	CAD	Plasma	Down		67 CAD; 31 healthy subjects	Fichtlscherer etal. (2010)
miR-17/92 cluster						
miR-155						
miR-145						
miR-135a	CAD	PBMC	Up		25 unstable AP; 25 stable AP; 20 healthy subjects	Hoekstra etal. (2010)
miR-147			Down			

* 
HF, heart Failure; AM, acute myocardial infarction; STEMI, ST-elevation myocardial infarction; proBNP, pro brain natriuretic peptide; BNP, B-type natriuretic peptide; CK-MB, creatine kinase isoenzyme MB; CAD, coronary arterial disease; PBMC, peripheral blood mononuclear cell; AP, angina pectoris.*

### CIRCULATING miRNAs IN HEART FAILURE

The first putative miRNA biomarkers in heart failure were discovered in a miRNA array on plasma of 12 healthy controls and 12 heart failure patients (Tijsen et al., 2010). From this array, 16 miRNAs were selected for a second clinical study in 39 healthy controls and in 50 cases with reports of dyspnea, of whom 30 were diagnosed with heart failure and 20 were diagnosed with dyspnea attributable to non-heart failure-related causes. In this study, 6 miRNAs (miR-423-5p, miR-18b-3p, miR-129-5p, miR-1254, miR-675, and miR-622) were elevated in patients with heart failure, with miR-423-5p positively correlated with NT-proBNP levels and most strongly related to the clinical diagnosis of heart failure. The increase of circulating levels of miR-423-5p could be confirmed by several other studies including hypertension-induced heart failure patients (Dickinson et al., 2013), systolic heart failure patients (Goren et al., 2012) and patients with dilated cardiomyopathy (Fan et al., 2013). In contrast, in patients with a reduced systemic right ventricular function and decreased ejection fraction, circulating miR-423-5p concentrations were not elevated, suggesting that miR-423-5p discriminates between sub-types of heart failure (Tutarel et al., 2011).

In a different study, the expression of 3 miRNAs in plasma of 10 heart failure patients and 17 asymptomatic control subjects was analyzed, demonstrating that the endothelium-derived miR-126 was negatively correlated with age, NT-proBNP, and New York Heart Association classification. Decreased miRNA-126 was also found in atherosclerotic coronary artery disease (CAD) and in patients with type 2 diabetes mellitus and may reflect the condition of vascular endothelial cells in heart failure patients (Fukushima et al., 2011).

Also plasma levels of several other miRNAs, including the heart-muscle enriched miRNAs miR-1, -133a, -208b, and -499; fibrosis-associated miRNAs miR-21 and miR-29b; and leukocyte-associated miRNAs miR-146, -155, and -223 were tested as candidate biomarkers (Corsten et al., 2010). This study demonstrated that in humans, diverse conditions of myocardial damage are associated with striking perturbations of plasma levels of cardiac specific miR-208b and miR-499 in acute heart failure (minimal), viral myocarditis (marked), and AMI (extensive). An intriguing observation was the correlation of miR-133a plasma levels with NT-proBNP in asymptomatic patients with diastolic dysfunction, which was not observed in acute heart failure patients.

### CIRCULATING miRNAs IN MYOCARDIAL INFARCTION

Plasma levels of miR-208b and miR-499 both have been highly associated with AMI. Also, it was demonstrated that measuring miR-1 in plasma is a good approach for blood-based detection of human AMI (Ai et al., 2010). Circulating miR-1 is significantly increased in the blood of AMI patients compared to non-AMI subjects and were positively correlated with serum CK-MB (creatine kinase-MB; Cheng et al., 2010). In a rat model of AMI induced by coronary ligation, serum miR-1 is increased early after AMI with a peak at 6 h, in which an increase in miR-1 of over 200-fold was demonstrated. Serum miR-1 returned to baseline levels at 3 days after AMI (Cheng et al., 2010). Also, increased miR-1 is well correlated with abnormal QRS complex widening (a reflection of abnormal electrical rhythm) in AMI, and after treatment, plasma miR-1 recovered to normal values (Ai et al., 2010). These data indicate that circulating miR-1 could serve as a biomarker for diagnosis of AMI and associated ischemic arrhythmias.

Next, an array analysis of miRNA production in various human tissues was reported, demonstrating that miR-499 was produced almost exclusively in the heart. To determine whether this miRNA could serve as a biomarker for cardiovascular diseases, the authors assessed the plasma concentrations of miR-499 in 14 individuals with acute coronary syndromes, 15 individuals with congestive heart failure, and 10 individuals without cardiovascular diseases. Plasma miR-499 concentrations were elevated in all AMI patients, but were below the detection limit in the other patient groups (Adachi et al., 2010).

Another miRNA microarray study demonstrated that miR-1, miR-133a, miR-499, and miR-208a were elevated in plasma from 33 AMI patients compared to as well as healthy subjects, patients with non-AMI coronary heart disease, or patients with other cardiovascular diseases. Notably, within 4 h of the onset of symptoms of the disease, miR-208a was easily detectable in AMI patients, but remained undetectable in non-AMI patients (Wang et al., 2010a). Also, circulating miR-133a levels were increased in 216 patients with ST-elevation myocardial infarction (STEMI), and associated with decreased myocardial salvage, larger infarcts, and more pronounced reperfusion injury (Eitel et al., 2012). In contrast, it has been reported that miR-133a levels were not associated with left ventricular remodeling or function after myocardial infarction, nor with BNP, excluding miR-133a as a useful biomarker for left ventricular remodeling after MI (Bauters et al., 2013).

Furthermore, it was reported that miR-30a, miR-195, and let-7b could be used as potential biomarkers for AMI (Long et al., 2012). The authors analyzed plasma samples from 18 patients with AMI and 30 healthy adults, and demonstrated that all 3 miRNAs reached their expression peak 8 h after the onset of AMI and these miRNAs showed significant diagnostic value for AMI using receiver operating characteristic curve analyses.

### CIRCULATING miRNAs IN ATHEROSCLEROSIS

Coronary artery disease is characterized by plaque formation along the inner wall of coronary arteries, which narrows the arterial wall and gradually restricts blood flow to the heart (Libby et al., 2011). In one study, circulating miRNA profiles in plasma from eight CAD patients and eight healthy subjects were assessed by a microarray approach. Validation of the obtained results in a larger patient cohort by qPCR revealed that circulating endothelial-associated miR-126, the miR-17/92 cluster, inflammation-associated miR-155 and smooth muscle cell-associated miR-145 were significantly reduced in CAD patients (Fichtlscherer et al., 2010).

Another study using real-time PCR-based profiling showed that among 157 miRNAs expressed in peripheral blood mononuclear cells of CAD patients, miR-135a and miR-147 were fivefold overexpressed and fourfold decreased, respectively. This study also indicated the possibility to discern unstable pectoris angina patients from stable patients due to their relatively high expression of circulating miR-134, miR-198, and miR-370, opening the possibility of a miRNA signature for patients at risk for acute coronary syndromes (Hoekstra et al., 2010).

The potential of circulating miRNAs as biomarkers for cardiovascular diseases is promising. Indeed extracellular miRNAs present many properties of ideal biomarkers, including their detection in many biological fluids, their stability in RNAse-rich body fluids, and their tissue-specific expression patterns. More efforts on much larger cohorts of patients with various cardiovascular diseases are needed to reach sub-stratification of patients. Another appealing outlook of extending available biomarkers is the possibility to perform network analyses and multi-marker biomarker panels for individual patients, allowing increased sensitivity in diagnostics or prognostics than can be expected from the assessment of a single biomarker (Eurlings et al., 2012), as evidenced by the analysis of distinct clusters of miRNAs associated with myocardial infarction in a large study of patients (Zampetaki et al., 2012). Next-generation sequencing is an opportunity for miRNA profiling efforts and for further discovery of new miRNAs in a determined biological or pathological situation (Lee et al., 2010; Lawless et al., 2013). Nevertheless, there are still technical limitations in studying extracellular miRNAs as biomarkers. No consensus has been obtained in terms of normalization methods nor the use of equal amounts of serum or plasma, or the use of spike-in controls or the use of housekeeping miRNAs yield wide-spread consensus (Kroh et al., 2010; Qi et al., 2012).

## Conflict of Interest Statement

The authors declare that the research was conducted in the absence of any commercial or financial relationships that could be construed as a potential conflict of interest.
